# Ingraining Polio Vaccine Acceptance through Public Service Advertisements in the Digital Era: The Moderating Role of Misinformation, Disinformation, Fake News, and Religious Fatalism

**DOI:** 10.3390/vaccines10101733

**Published:** 2022-10-17

**Authors:** Qiang Jin, Syed Hassan Raza, Muhammad Yousaf, Rehana Munawar, Amjad Ali Shah, Saima Hassan, Rehan Sadiq Shaikh, Emenyonu C. Ogadimma

**Affiliations:** 1 Intercultural Communication Research Center, Hebei University, Baoding 071000, China; 2 Institute of Media and Communication Studies, Bahauddin Zakariya University, Multan 66000, Pakistan; 3 Centre for Media and Communication Studies, University of Gujrat, Gujrat 50700, Pakistan; 4 Department of Mass Communication, National University of Modern Languages, Islamabad 44000, Pakistan; 5 District Headquarter Hospital, Multan 66000, Pakistan; 6 Centre for Applied Molecular Biology, University of the Punjab, Lahore 54000, Pakistan; 7 College of Communication, University of Sharjah, Sharjah P.O. Box 27272, United Arab Emirates

**Keywords:** polio vaccine, vaccine acceptance, public service advertisement, protection action decision model, misinformation, fake news, disinformation, religious fatalism

## Abstract

Recently, misinformation and disinformation, as well as fake news, have become global threats to public health owing to their role in spreading viral health hazard information. The growing explosive religious fatalistic views presented on social media and widespread misinformation, disinformation, and fake news can result in detrimental outcomes in adopting protective behavior. The moderating implications of misinformation and religious fatalism can be severe, leading to adverse effects on polio vaccine acceptance. Consequently, this research provides brief empirical evidence on the efficacy of risk communication strategies to address polio vaccine reluctance in a digital age landscape, an area that remains understudied. This research argues that the spread of misinformation, disinformation, fake news, and religious fatalism is not solely the bane of the polio vaccine, but rather represents the absence of risk communication strategies. The study opines that polio vaccine acceptance can be improved using risk communication strategies. Recognizing these risk factors and counter-risk communication strategies, this research tested a theoretical model using the cross-sectional survey design. Overall, data was collected from 2160 parents with children aged below five years. The results, based on structural equation modeling, revealed that public service advertisements are an effective tool to counter the inverse impacts of misinformation, disinformation, fake news, and religious fatalism. Furthermore, the inverse moderating role of misinformation, disinformation, fake news, and religious fatalism has been verified to potentially diminish polio vaccine acceptance. These results suggest that healthcare providers must identify and address all forms of digitally disseminated information that encumbers public health behaviors. Accordingly, this research recognized the utilization of evidence-based strategic communication campaigns to cultivate and encourage the literacy necessary to counter health hazard information, including misinformation. This study’s findings will benefit health and other concerned authorities in utilizing strategic communication on different media platforms to reduce or eradicate the polio endemic.

## 1. Introduction

Polio is a disabling and deadly disease that causes nerve harm and paralysis in children [1]. Out of every 200 reported polio cases, one case leads to a permanent disability, with a 5–10% mortality rate among paralytic persons [2]. In the last century, polio remained a life-threatening epidemic that caused paralysis among millions of children [3]. As a result of this gloomy narrative, the Global Polio Eradication Initiative (henceforth GPEI) was launched in 1988 to combat the causes of polio spread and improve worldwide polio vaccine acceptance. Since the establishment of the GPEI, there has been a rapid decrease in the reported polio cases. Over the past 30 years, wild poliovirus cases have significantly waned by 99%. The reported polio cases at the inception of GPEI in 1988 were 350,000 across 125 countries [4]. These cases were reduced to 33 reported cases (of polio) in 2018. With the joint efforts of the World Health Organization (henceforth WHO) and the GPEI, poliovirus type 2 was eradicated in 1999. Similarly, the American region (in 1994), the Western Pacific Region (in 2000), and South-East Asia Region (in 2014) were declared polio-free regions by WHO [5]. Given the global concern and initiatives aimed at eliminating polio, many governments and private organizations have contributed to the polio eradication drive through worldwide polio vaccination campaigns. For example, since the establishment of the GPEI, roughly USD 17 billion has been provided to support the polio eradication drive [6]. As a result of these tremendous efforts made by the global community, a rapid decline in polio cases was observed in 2019, as WHO reported that the polio vaccine had saved approximately 1.5 million children [7].

The collaborative initiative of the GPEI and WHO in both global and local communities effectively eliminated polio cases using different strategic means, including; (1) surveillance, (2) communication campaigns, and (3) substantial immunization programs in most parts of the globe. Unfortunately, a few countries, such as Pakistan and Afghanistan, have remained endemic [8]. In 2022, the GPEI categorized eight countries, including Pakistan, as at risk of an outbreak or epidemic. While Pakistan actively participated in the polio eradication drive and supported efforts of the GPEI and WHO, it is still struggling with this issue and has not yet become a polio-free country. In the fight to eliminate polio, the government of Pakistan donated around USD 387 million to the GPEI through financial assistance from the Islamic Development Bank and USD 121 million with financial aid from the Japan International Cooperation Agency. Pakistan also received financial aid of USD 58 million from developed nations to support GPEI efforts for polio eradication from 1985 to 2019 [2]. Despite these huge efforts, the number of polio cases increased progressively, from 53 cases in 2012, to 93 cases in 2013, up to 307 cases in 2014. This was the country’s highest number of polio cases recorded in recent years. However, in the past few years, the polio eradication drive has made good progress, given the massive polio vaccination campaigns, which have brought about a gradual decrease in the number of cases reported in Pakistan from 2015–2018. For example, 54 cases were reported in 2015, 20 in 2016, 8 in 2017, and 12 in 2018. Conversely, 2019 and 2020 again saw a rise in cases, i.e., 147 in 2019 and 84 in 2020 [9,10]. The rising polio cases reported in Pakistan are alarming, compared to the rates in the rest of the world. For example, in 2014, out of the 359 polio cases reported globally, Pakistan had a whopping 306 cases. [11]. These are disturbing facts because a nation that is still endemic can become the source of the outbreak for other nations. The GPEI listed 33 potential outbreak countries that have eradicated local poliovirus cases; but may still be confronted with re-infection. These re-infections were forecasted mainly because of the possible importation of the poliovirus from endemic countries. Therefore, there is a dire need to unpack the issues regarding the existence of the poliovirus in endemic countries such as Pakistan [9], where there seems to be polio vaccine resistance among some regions.

Previous literature has identified that polio vaccine reluctance persists in a few regions of Pakistan [9]. The literature has also revealed that Pakistani authorities have made longstanding efforts to combat polio. However, Pakistan remains endemic owing to the local communities’ misperception and implicit bias over Pakistan’s collaborative efforts with the global community [12]. Apart from this particular threat, WHO has also identified various obstructions in eradicating poliovirus, including disinformation emanating from religious beliefs and the social media-driven overabundance of misinformation and fake news based on myths about the polio vaccine in Pakistan [2]. In recent years, Pakistan has also witnessed several issues that hinder polio eradication initiatives, such as parental denial of polio vaccination, myths, misinformation, and religious factors. Together, these factors remain the bane of the steady progress of attaining the status of a polio-free Pakistan [11]. Pakistani media has also reported several unfortunate incidents that have hindered polio vaccination campaigns. For example, based on myths and fabricated information, local communities have resisted polio vaccination campaigns. Health facilities have also been attacked because social media fabricated death claims due to the polio vaccine [9].

This study intends to probe into the forces militating against polio vaccination. In doing so, we integrated misinformation, disinformation, fake news, religious fatalism, and fanaticism, which have been identified as the threatening factors in developing protective health behavior, with the protective action decision model (henceforth PADM). Several studies have advocated mass media-driven target campaigns to counteract misinformation and fabricated religious beliefs [13]. However, these communicative efforts must align with the socio-psychological mechanism explained by past theories that describe the health-related decision-making process [14]. Researchers have long been interested in explaining how individuals react to environmental cues or socially communicated alerts about health threats. This study intends to underpin the phenomena of adaptive protective behavior related to polio vaccination.

Further, this research extended PADM by conceptualizing public service advertisements to find out how digital media platforms are influencing the efforts of polio eradication campaigns. This study, consistent with theories on social influence, persuasion, behavioral decision-making, attitude–behavior interactions, protective action, and innovation processes, offers valuable suggestions on how risk communication might impact immediate disaster reaction and long-term hazard modifications. According to scholars, communication contents can effectively diminish risks implanted by myths by inducing a greater extent of risk perceptions, promoting the resource (polio vaccine) perception, and instilling greater trust in the activities of the health authorities [15]. We argue that people are now living in the digital and social media era and are vulnerable to fake and fabricated information hindering their adaptive health behavior.

Therefore, considering and integrating psychological, social, risk communication, and digital communicative elements can reveal a more comprehensive framework to understand the efficacy of communication campaigns in real-world scenarios. Given the gap in literature in this area, we aimed to boost the modest efforts that have been so far exerted in exploring the integrated negative effect of misinformation and religious fatalism on health authorities’ efforts toward eradicating poliovirus. For example, researchers have studied the psychological characteristics most closely associated with healthcare views and practices for years. The role of religious fatalism in shaping health outcomes remained a particular focus. Similarly, in the context of COVID-19, misinformation and infodemics have been widely studied. However, the moderating influence of religious fatalism and misinformation have mainly remained unexplored in the context of polio eradication campaigns.

The informational support provided by commination campaigns serves as the external cues to improve the chances of the health protection actions such as (accepting) the polio vaccine. Furthermore, the role of public service advertisements has been proven to promote better behaviors, including favorable changes in the perception of individuals [16]. The essential aspects of the obstacles to public service advertisements—psychological and communicative factors—have been combined to propose a more comprehensive framework based on PADM. The research advances understanding of the way in which these factors affect people’s adoption of protective measures. As a result, the purpose of this paper is to come up with an updated version of PADM by combining three applications: (a) the creation of risk communication programs, (b) the use of evacuation modeling, and (c) the adoption of long-term hazard modifications.

## 2. Literature Review

### 2.1. Protective Action Decision Theory

Lindell and Perry [17] proposed PADM, in which they presented tenets to understand people’s risk adaption behavior in crises and risky circumstances, including natural disasters and environmental hazards. However, Lindell and Perry [17] subsequently modified and extended PADM by adding new constructs to tap the public’s behavioral responses during hazard-related situations. PADM was developed in light of studies of human reactions to natural catastrophes and threats [18]. The extended PADM combines the analysis of social and environmental cues with the interpretation of messages from social sources (sent through communication channels) to identify and mitigate potential threats. PADM emphasizes three vital pre-decision processes—exposure, attention, and interpretation of environmental/social cues, or the receipt, attention, and understanding of warnings [19]. Decisions regarding how to react to an immediate or long-term danger are grounded in the updated model’s identification of three critical perceptions: threat perception, protective action perception, and stakeholder perception. A person’s actions are influenced by their choice of protective measures and the opportunities and challenges presented by their immediate environment. This research details the new model and the studies that inspired it and discuss three unanswered practical questions highlighted and established as research priorities.

**Figure 1:**
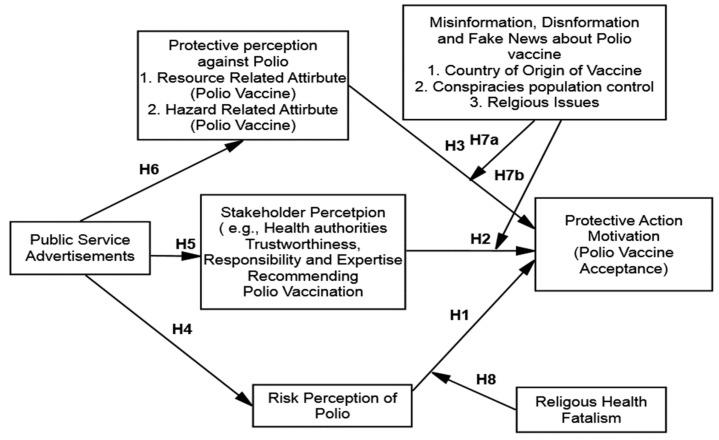
Conceptual model of the study.

PADM, risk communication, and the information processing model provide a comprehensive framework that explains the role of information in developing protective behavior. PADM postulates that individuals exposed to numerous hazards or adversities can obtain cautionary information from external sources, such as the media [20]. Information containing warnings and hazard-related cues underwrites the development and realization of individuals’ risk perception, which leads to protective behavioral responses [15]. PADM has been widely employed to explore various risk-related domains, such as health [19], natural disasters [20], and environmental awareness [21]. Therefore, this study applied the PADM framework to examine the adoptive behavior of polio vaccination. This research postulated a conceptual model (see Figure 1) based on the tenets of PADM that are characteristically developed to understand risk and hazard-related scenarios. Consequently, this research has extended PADM and integrated the contextual factors that can enhance the explanatory power of the PADM in the context of the digital media landscape. PADM is delineated in the following sections in greater detail.

#### 2.1.1. Risk Perception

Perceived risk is a key variable in PADM that predicts people’s conduct in risky circumstances. In relation to polio, it is assessed by expectations about the possibility of personal bodily harm, as well as the social impacts induced by the polio vaccine [22]. It is widely agreed that people’s perceptions of poliovirus hazards are mostly comprised of likelihood and consequences. However, specific well-known methods of perception incorporate risk traits such as fear and unknown risks. This seems to include people’s anticipation about the personal effects of polio vaccination, which adds another dimension to their overall danger. Loss of life, physical harm, property damage, and the inability to go about one’s routine (including going to work, school, or conducting business) are only some of the predicted individual ripples. In addition, studies conducted on polio vaccine hazards and other diseases have prompted modifications to the polio vaccination and its long-term consequences [22].

Research on protective responses to polio vaccination and other risks has been conducted [20]. Such studies were effective in describing people’s reactions to a wide variety of technology and social activities (especially adoptive behaviors). However, the connection between these concepts and how individuals perceive and react to polio vaccine concerns is not immediately apparent. Studies on polio vaccines and catastrophes have focused on defining research connected to the perceived risk regarding vaccine safety [8], which has increased significantly over the last several decades. It is noted that perceived risk is essential in domains stretching from psychology to public health [23]. Public perceptions about evolving phenomena (for example, vaccination) are influenced mainly by perceived risk [24]. Studies confirmed that perceived risk is the primary factor driving people’s behavioral intentions to adapt to numerous dangers. In addition, the literature further confirms the substantial influence of people’s risk perception on the acceptance of vaccination, such as COVID-19 and polio vaccines [25]. Available evidence shows an inverse relationship between the public perceptions of risk and their actions toward vaccine safety [26]. Based on past theoretical and empirical works, we conclude that public aversion to vaccination increases as people become aware of and weigh their hazards owing to misinformation, disinformation, and fake news. Stakeholders’ perceptions of authority (local governments and provincial or national television stations, in this study) and experts’ credibility are crucial in determining their propensity to take preventative action; thus, we hypothesized that:

**Hypothesis** **(H1).** *Risk perception positively influences polio vaccine acceptance*.

#### 2.1.2. Stakeholder Perception

Another antecedent to the protection actions is stakeholder perception, which refers to individuals’ anticipation about the extent of the expertise, reliability, and protection responsibility of the stakeholder [27]. The polio eradication initiatives are primarily promoted by the health authorities, including global organizations and local governments, which are regarded as the primary stakeholders. Therefore, positive perception is crucial to promote positive health behavior, such as polio vaccination acceptance. It is believed that parents with more trust in these stakeholders’ recommendations would likely have less resistance to accepting the polio vaccine. Specifically, risk communication might benefit significantly from a greater emphasis on the credibility of information obtained from authorities and experts [28]. Accordingly, stakeholder perceptions of various information sources might motivate public intent to take precautionary measures [29]. Our research is based on the premise that individuals may learn about polio vaccinations from various sources, including newspapers, the internet, television, and advertisements. Each person’s assessment of the material’s credibility may vary, depending on the source. When people have a strong impression that the information is reliable, they are more likely to adopt the precautions that have been suggested. Therefore, we proposed that:

**Hypothesis** **(H2).** *Stakeholder perception positively influences polio vaccine acceptance*.

#### 2.1.3. Protective Action Perception

Protective action perception, stakeholder perception, and risk perception are the three aspects of perception that should be considered when deciding whether to adopt polio hazard adjustment activities [17]. There are two primary aspects to consider when forming an opinion on what precautions should be taken: the potential threats and the available resources. Polio vaccination-associated traits suggest that applying preventive steps will mitigate the risks and that people can make such efforts, highlighting the link between risk and hazard adjustment [30]. Attributes associated with polio vaccines may represent an individual’s perceived capacity to protect people and property and the perceived value of hazard modifications. Furthermore, PADM reveals that when individuals perceive more significant polio vaccine-related qualities, they may be more confident in completing preventive activities, contributing to adoption intentions and actual adoption behaviors [31]. People may be less likely to make necessary alterations to risks when they believe the cost of taking precautions would be much greater than it is because of increased resource demand. According to the findings of PADM, the correlation suggests that low rates of polio vaccination uptake and actual behavioral risk adjustments are related to perceptions of significant resource demands. Following this logic, we hypothesized the following:

**Hypothesis** **(H3).** *Protective action perception positively influences polio vaccine acceptance*.

#### 2.1.4. Public Service Advertisements

Apart from the socio-psychological and environmental factors, PADM also proposes informational elements that influence the individual perceptual mechanism involved in developing protective actions [17]. PADM noted that individuals receive actionable cues from exposure to risk information. The three perception-based mechanisms, such as risk perception, are directly influenced by exposure to risk information communicated through different sources [17]. PADM posited that the communicative environment is vital in outlining the protective mechanism, calling it the pre-decision process. PADM further delineated the information processing stage and identified several environmental, informational, and social cues that people receive. Moreover, the message characteristics, such as source, channel, and content, are also acknowledged by PADM. Together, these communicative environmental factors determine people’s responses, which are influenced by; (1) exposure, (2) attention, and (3) comprehension. Once people encounter information from environmental or social cues obscuring several information attributes, these three pre-decisional processes will likely develop a protective outcome. For example, information regarding polio threat can give warning cues and enhance the realization of the health hazard among people [32]. This research sought to highlight the effectiveness of the public service messages in outlining the protective processing (e.g., risk perception) that can further influence the protective action stage.

Past psychological theories, such as the health belief model, also highlighted the role of media in health-related issues and defined the media as an external cue that determines health behavior [33]. Public service advertisements are the most commonly employed media content in health communication campaigns. Public service advertisements usually underscore informational and environmental verbal and non-verbal cues [16]. To this end, these advertisements are designed to capture the audience’s attention through repeated exposure to intensive health communication campaigns from different media outlets. Past studies have established that the verbal and non-verbal cues used in public service advertisements indeed facilitate an individual’s deliberation and comprehension [16]. Therefore, public service advertisements trigger one’s cognitive and affective mechanisms. This ability of public service advertisements is well-known in the persuasion literature. For instance, non-verbal cues (ad appeals, such as scarcity) can influence risk perception and thus facilitate protective actions. This also applies to message framing, ranging from persuasion to motivational protective behaviors, in media, especially in public service advertisements [34,35,36]. The manifested messages and exposure lead to developing information behaviors, such as individuals developing an interest in searching for information provided by health authorities (stakeholders) and thus realizing the intensity of the problem (risk perception). Likewise, media campaigns, such as public service advertisements, are a tool for strategically managing hazards by providing resource-related information [37]. For example, information about the appropriate solutions to the threat of polio may be children’s polio vaccination. This information can be effectively communicated through public service advertisements. Therefore, a well-designed public service advertisement can manipulate the targeted protective actions.

Ample literature has also identified the influence of public service advertisements on protective health responses, such as risk perception and vaccine benefits [35]. These studies provide empirical support regarding the influence of public service advertisements on facilitating the protective behavior mechanism by informing the people of the facts [16,35]. Furthermore, studies highlighting the health benefits of vaccination described in communication campaigns affirmed that communication could positively influence protective actions by developing a positive perception. Similarly, the literature has reported the capability of media content to increase risk awareness; thus, it can effectively provide threat cues [38]. Further, research on public service advertisements also validated that people can encode health messages by paying attention once exposed to them. The information processing from advertisements can facilitate the elaboration process, causing people to deliberate. If they anticipate that the information source is reliable, there is a greater chance of persuasion. Therefore, exposure to public service advertisements can instill the protective action perception; hence, people may perceive the polio vaccine as a resource and hazard attribute to evade the threat of polio among their children. Based on the above prognosis, it is expected that public service advertisements will be effective in enhancing risk, stakeholder, and protective action perception; thus, it is hypothesized that;

**Hypothesis** **(H4–H6).** *Public service advertisements positively influence risk perception (H4), stakeholder perception (H5), and protective action perception (H6).*

#### 2.1.5. Misinformation, Disinformation, and Fake News

The term misinformation refers to incorrect and unverified misleading information. Misinformation is distinguished from disinformation, which is intentionally misleading [39]. On the other hand, fake news represents the shared features of misinformation and disinformation [40]. However, fake news is different from these, as it presents incorrect and misleading information as news. The literature indicates that unverified misinformation, disinformation, and fake news regarding immunization and vaccination can spread quickly and maliciously on digital platforms [41]. Research has also verified that the interactive nature of digital media generates a powerful forum for disseminating myths, lies, and falsehoods about vaccine speculations [41,42]. According to these studies, social media is routinely used to propagate erroneous and imprecise information about vaccines. Other studies have identified that social media facilitates anti-vaccine propaganda and conspiracy theories [43]. Therefore, it is pertinent to assert that the digital media landscape in recent times has been profoundly challenging in outlining positive public health responses [44]. This is because digital platforms facilitate unchecked, unverified, and user-generated content that may impact public vaccination decisions. In this way, misinformation and disinformation can affect implicit negative beliefs about the polio vaccine and promote risk perception [9].

Moreover, numerous studies have shown that misinformation could hinder protective actions and negatively influence one’s motivation to engage in protective behavior [45]. This research postulated that the misinformation and disinformation available online could determine the strength of the relationship between the stakeholder and protective perceptions regarding the acceptance of polio vaccination. For example, those individuals who perceive the stakeholders or resources (i.e., the polio vaccine) as reliable and trustworthy would be less vulnerable to misinformation. Thus, exposure to misinformation or disinformation impacts the relationship between protective measures, trust in stakeholders, and the polio vaccine. Likewise, regarding hazard attributes, people may start thinking they are less vulnerable to polio due to exposure to misinformation or disinformation. Therefore, there is the possibility that misinformation or disinformation diminishes the realization of the value of resources (polio vaccine) and stakeholder efforts to promote protective actions; thus, we hypothesized that;

**Hypothesis** **(H7).** *Misinformation, disinformation, and fake news inversely moderate the relationship between stakeholder perception (H7a) and protective action perception (H7b) regarding polio vaccine acceptance (protective action motivation).*

#### 2.1.6. Religious Fatalism

Religion and fatalism, which we call “religious fatalism,” are two concepts that have received little attention from academics. Fatalism, which refers to the belief that an individual’s health result is planned or designed solely by a greater authority and is beyond the individual’s control, has been studied as a hindrance to participation in health awareness campaigns and health care consumption. Fatalism views a person’s health as decided by external factors such as luck, destiny, or God, but not as something that can be actively improved. Health psychology researchers have pioneered studies in fatalism. Researchers have shown that African American women who adopt fatalistic views are less likely to participate in preventive care [46]. Past research on religious fatalism showed that people downplayed the seriousness of their diseases by refusing treatment and relied on their religious beliefs to cope and as an excuse for not following medical advice [46,47]. It has also been shown that people are sometimes more likely to have fatalistic beliefs about cancer if they are older, have lower incomes, have less education, and have inadequate access to health care [47].

This research uses the term “religious fatalist” to describe those whose fatalism is heavily influenced by their spiritual or religious activities [48]. This does not imply that all “religious” people are fatalists or that religious faith is the sole source of fatalism. In contrast, this research aims to look at the beliefs that may link the psychological domain of control components not captured by fatalism assessments with the religious/spiritual beliefs and practices embraced by faith groups. Although many people who identify as religious may not have such beliefs, the possibility remains that some may raise the issue of how such beliefs may influence health-related choices and actions of those who adopt them [46]. Some studies have shown that the consequences of religious faith and fatalism on an individual might vary significantly [48]. Researchers have argued that in addition to the well-established benefits, certain religious beliefs may prevent people from using healthcare services, and some healthcare practices might result in unfavorable health results [49]. Religious beliefs and practices can clash with medical experts’ recommendations and negatively impact a patient’s health, leading to misconceptions in patient awareness and non-compliance with prescribed treatments. The current research argues that the extent of religious fatalism could diminish the perceived level of the risk. Therefore, people may feel that their children are not vulnerable to polio disease and feel less at risk. In turn, vaccine acceptance would be low among these groups, and we hypothesized that;

**Hypothesis** **(H8).** *Religious fatalism inversely moderates the relationship between risk perception and polio vaccine acceptance*.

## 3. Materials and Methods

### 3.1. Design, Participants, and Procedure

This research employed a cross-sectional survey to validate the proposed model. The core questions raised in the study focused on the influence of public service advertisements in facilitating polio vaccine acceptance and moderating the role of misinformation, disinformation, and fake news. We targeted Pakistan’s married population with children below five years to verify this information processing mechanism. Owing to the nature (and resources) of data collection, we used the multistage sampling method to select the appropriate sample. The sampling procedure was based on three stages. In the first stage, a list of Pakistan’s districts was obtained, and eight districts were randomly selected using the excel program R. In this procedure, equal representation to each province of Pakistan was ensured, and two districts were selected from each province. In the second stage, a list of smaller administrative units, known as “tehsils,” was procured from the eight chosen districts. Next, tehsils from each district were randomly selected using the lottery method of random sampling. In the third stage, a purposeful sampling technique was adopted to ensure the appropriate data collection from the targeted population living in these eight areas.

This research selected the married population of Pakistan with children below five years and provided representation from all provinces (e.g., administrative units) of Pakistan. This selection criterion aligns with the aims of this research. It is rationalized based on the following justifications: (1) the sampling technique ensured the representation of all administrative units (e.g., provinces), (2) multistage sampling was used to randomly select the areas, while purposeful sampling ensured the selection of desired participants (as well as a large number of participants), (3) this type of sampling was used to determine whether cases of polio were reported or the polio virus was found across all provinces of Pakistan over the last several years.

Furthermore, before executing the data collection procedure, a G-power analysis was conducted to determine the suitable sample size for this study. The study involved six antecedents and one dependent variable, and the results revealed that the 1800 sample size is appropriate. Data were collected for four months, from May 2022 to August 2022, with the assistance of professional data collectors affiliated with a data collection firm. The research team provided a one-day training to the data collectors to clarify this research’s aims and ethical values. Overall, data was collected from 2500 participants. However, 2160 completed responses were considered for this research. The data collection team approached the participants and obtained ethical, informed consent before requesting that they fill out the questionnaire to tap the relevant variables of interest.

### 3.2. Instrumentation

The variable of public service advertisement was measured using three-item scales from the modified version of the health media exposure construct by Tan and Hornik (2014). The constructs of risk, stakeholder, and protective action perception were measured using modified scales adopted from the literature [17,18]. The risk and the stakeholder perceptions were measured using three items for each construct. In comparison, protective action perception was measured using six items with two dimensions of resource and hazard-related attributes. Religious fatalism was measured using the four items adapted and modified from the work of Franklin [46]. The study operationalized protective action motivation as polio vaccine acceptance, and this was measured using the three items adapted from the literature [50,51]. Lastly, misinformation, disinformation, and fake news were measured using three modified representative items regarding media attention [52].

## 4. Results

### 4.1. Demographic and Preliminary Analysis

Demographic analysis was conducted after cleaning and eliminating missing responses. Thus, the study proceeded with the 2160 usable responses. The investigation revealed 1393 (64.5%) males and 767 (35.5%) females in the sample. Regarding the provincial ethnic groups, the study outcomes significantly resemble those of the Pakistani national census. Overall, 1128 (52.2%) respondents were from Punjab, 536 (24.8 %) were from Sindh, 422 (19.6%) were from Khyber Pakhtunkhwa, and 74 (3.4%) were from Balochistan. Hence, the parents’ sample adequately represented the real ethnic composition of Pakistan. Education-wise, the analysis revealed that 871 (40.3%) respondents were matriculated, followed by 581 (26.8 %) who were college diploma or degree holders, 397 (18.4%) were illiterate or below matriculation level, and 311 (14.5%) held master’s/above master’s degrees. Complete responses were entered in the SPSS 22.0 software, and the normality of the data was examined. We employed the skewness/kurtosis tests and a visual examination of the outliers. After deleting the outlier cases from the 2160 valid responses, we proceeded with the primary analysis, including a correlation analysis, with 2013 responses. The results revealed a satisfactory level of correlation, as assumed in the proposed model (see Table 1), and the study proceeded for further analysis.

### 4.2. Measurement Model

This study used structural equation modeling (henceforth SEM), which is a suitable approach for discovering and analyzing complex multivariate data. SEM was used to examine the viability of the proposed causal model and to evaluate our model. SEM can determine how well a theoretical or conceptual model fits a dataset. Given that the sample size was more than 2000, the researchers used covariance-based SEM. The analysis of moment structures (henceforth AMOS) software estimate parameters was found using the maximum likelihood technique. In this study, we utilized AMOS 23.0 to estimate the parameters. Initially, the model fit was evaluated using the following criteria, as suggested by Kline: 2/df (the chi-square value divided by the number of degrees of freedom in the model) 3, RMSEA (root mean square error of approximation) 0.06, SRMR (standardized root mean residual) 0.10, Tucker–Lewis Index (TLI) > 0.9, and comparative fit index (CFI) > 0.90 [53]. We assessed build reliability using Cronbach’s alpha. The proposed model was tested using the constructs’ reliability and validity. Cronbach’s alphas were greater than the minimum acceptable value of 0.70. Consequently, there was satisfactory dependability across the board. To ensure the accuracy of our model, we checked it for convergent, discriminant, and content validity. We looked at factor loadings, composite reliability, and average variance extracted (AVE) to verify convergent validity.

**Figure 2:**
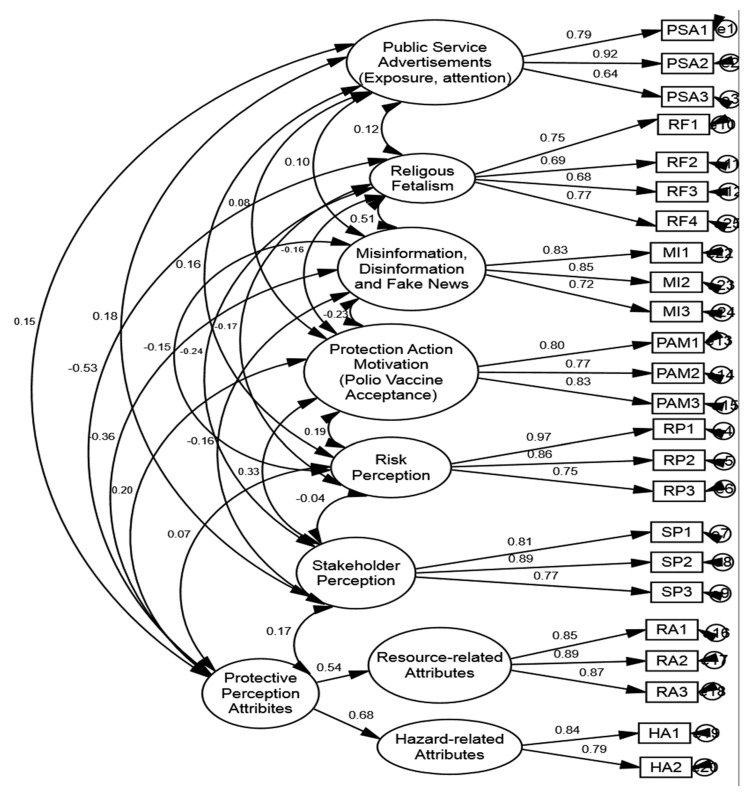
Measurement model.

The CFA findings (Table 2 and Figure 2) demonstrated that all factor loadings were greater than 0.6, the cutoff, except for one item of PPA, which was removed. The composite reliability was greater than the threshold value of 0.7, ranging from 0.803% to 0.963%. [54]. In addition, the AVEs for all constructions were greater than the 0.5 threshold value, falling in the range of 0.581 to 0.868. These findings suggested that our measuring approach has high convergent validity. The square roots of the AVEs across all constructs and inter-construct correlations should be compared to verify discriminating validity [55].

According to Table 3, our measuring model shows complete discriminant validity, since the square roots of the AVEs for each component are larger than the correlations between them, thus justifying the credibility of this study. All measures of model fitness (2 = 339.427, df = 152, 2 /df = 2.233; TLI = 0.976, CFI = 0.943; RMSEA = 0.046, SRMR = 0.045) pointed to an excellent match between the measurement model and the dataset. According to the CFA, all of these requirements have been met. Additionally, indicator items within each measurement scale were strongly linked to their theoretical foundations.

### 4.3. Hypothesis Testing

**Figure 3:**
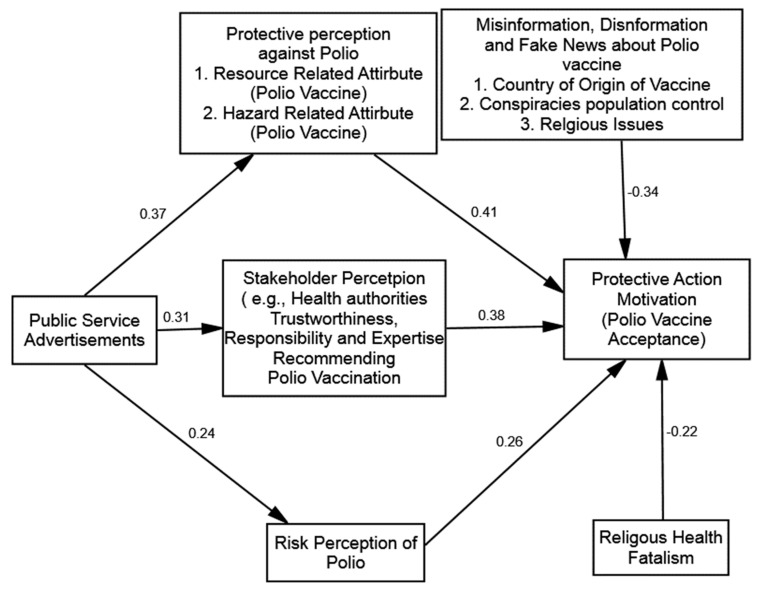
Structural model.

This research used the path analysis using AMOS 23.0, with the bootstrapping technique with 500 iterations, for testing the hypotheses. The research proposed three hypotheses delineating the direct influence of risk perception (H1), stakeholder perception (H2), and protective action perception (H3) on polio vaccine acceptance (protective action motivation). The results revealed that risk perception (β = 0.26, *p* = 0.001), stakeholder perception (β = 0.38, *p* = 0.001), and protective action perception (β = 0.41, *p* = 0.001) significantly and positively influenced polio vaccine acceptance (protective action motivation). Thus, H1, H2, and H3 were correspondingly accepted (see Figure 3).

The research also proposed three hypotheses delineating the direct influence of public service advertisements on risk perception (H4), stakeholder perception (H5), and protective action perception (H6). The results revealed that public service advertisements significantly and positively influence risk perception (β = 0.24, *p* = 0.001), stakeholder perception (β = 0.31, *p* = 0.001), and protective action perception (β = 0.37, *p* = 0.001). Thus, H4, H5, and H6 were correspondingly accepted.

### 4.4. Moderation Analysis

**Figure 4:**
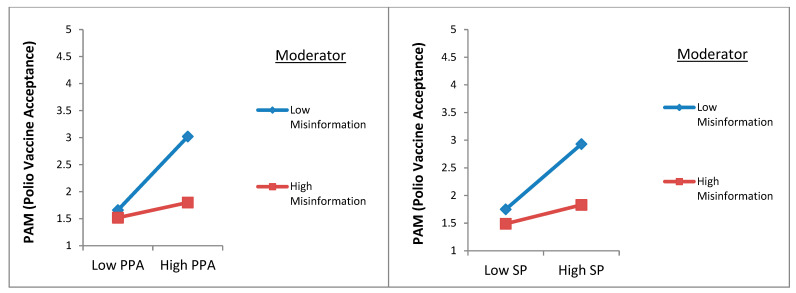
Moderation of misinformation.

This study posited an inverse moderation of misinformation, disinformation, and fake news in the relationship between stakeholder perception (H7a) and protective action perception (H7b) with polio vaccine acceptance (protective action motivation). The researchers used a bootstrapping technique on AMOS 23.0 to examine the three moderating hypotheses, while the two-step approach was used for moderating analysis. We added the interaction terms by estimating the standardized values of the variables of misinformation, disinformation and fake news, stakeholder perception, and protective action perception on SPSS 22.0. Next, the interaction terms were added to the SEM to compute the possible moderating effect. The results revealed that the moderating influence of misinformation, disinformation, and fake news was found to be inverse and significant in the relationship between stakeholder perception (β = −0.27, *p* = 0.012) and protective action perception (β = −0.21, *p* = 0.031) with polio vaccine acceptance (protective action motivation). Therefore, H5a and H7b were supported (see Table 4). Furthermore, the Dawson test was also used to verify these results graphically. The results of the Dawson test using Excel, presented in Figure 4, revealed that misinformation dampens the positive relationship between SP, PPA, and PAM (polio vaccine acceptance).

**Figure 5:**
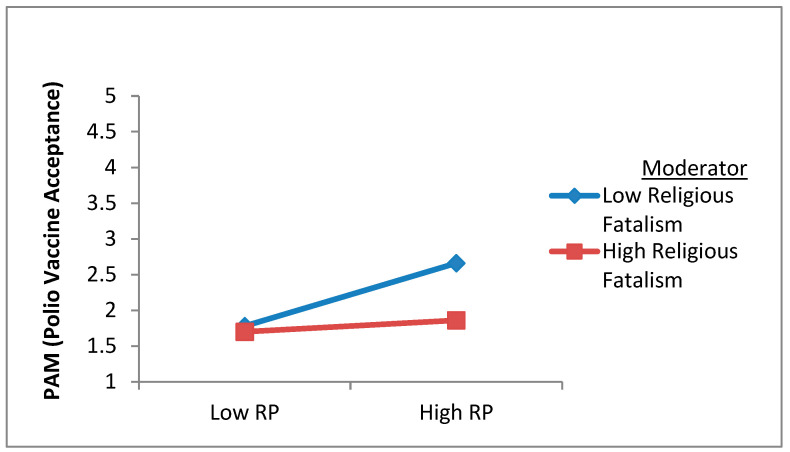
Moderation of religious fatalism.

This research also postulated an inverse moderation of religious fatalism in the relationship between risk perception (H8) and polio vaccine acceptance (protective action motivation). Using a similar two-step approach, we computed the interaction terms (Religious Fatalism X Risk Perception) by estimating the standardized values of the variables on SPSS 22.0. After the addition of the interaction term, the results revealed that the moderating influence of religious fatalism was found to be inverse and significant in the relationship of risk perception (β = −0.27, *p* = 0.012) with polio vaccine acceptance (protective action motivation) and, therefore, H8 was supported (see Table 4). Furthermore, the Dawson test was also used to verify these results graphically. The results of the Dawson test using Excel, presented in Figure 5, revealed that religious fatalism dampens the positive relationship between risk perception and polio vaccine acceptance (protective action motivation).

## 5. Discussion

This study used a cross-sectional design vis-à-vis a survey method to investigate the influence of public service advertisements in facilitating polio vaccine acceptance. Additionally, the moderating role of digital platforms in disseminating polio-related misinformation, disinformation, fake news, and religious fatalism among the general public in Pakistan was also evaluated. The study posed eight hypotheses that were supported. The findings of hypothesis one indicated that risk perception positively influenced polio vaccine acceptance. This finding implied that a person with a higher perceived risk of falling prey to polio was more willing to take the polio vaccine. Put differently, threat appraisal corresponds to an increase in vaccine willingness. Likewise, H2 posited that the perceived credibility and trustworthiness of the stakeholders recommending the polio vaccine encourages polio vaccine acceptance among the public. The higher the level of expertise, reliability, and credibility of the local and foreign authorities, the higher the acceptance level of the polio vaccine among the audience. In other words, parents who had more trust in the recommendations of these stakeholders would likely have less resistance to accepting polio vaccine programs. Given this projection, this finding supports H3 and proves that perceived protective action mitigates the risks and increases polio vaccine intake [30]. This finding supports the results of previous literature suggesting that confidence in the polio vaccine boosts public trust and less alteration regarding the polio vaccine acceptance [56].

Hypotheses (H4–H6) suggest that media campaigns in the form of public service advertisements positively influence risk perception (H4), stakeholder perception (H5), and protective action perception (H6). This proves that the media surveillance function creates awareness regarding risk and perceived threats (see Figure 2). These cues correspond to behavioral changes that facilitate protective behaviors among the public, thus increasing the willingness to get the polio vaccine [57,58]. Thus, it can be averred that public service messages create and encourage awareness among the public regarding the perceived risks that correspond to more favorable attitudes towards health-related behaviors, such as the acceptance of the polio vaccine [35]. The cues used in messages in public service advertisements can activate one’s deliberation and comprehension about the benefits of the polio vaccine [16]. Consequently, public service advertisements trigger one’s cognitive and affective mechanisms. Message framing ranges from persuasion to motivational protective behaviors in the media, especially in public service advertisements [34]. Therefore, exposure to public service advertisements can instill protective action perception that will encourage people to perceive the polio vaccine as a resource and hazard attribute to evade the threat of polio among their children.

Hypothesis H7 states that misinformation, disinformation, and fake news inversely moderate the relationship between stakeholder perception (H7a) and protective action perception (H7b) with polio vaccine acceptance (protective action motivation). The findings of this study support the belief that misinformation, disinformation, and fake news inversely moderate the relationship between stakeholder perception (H7a) and protective action perception (H7b) with polio vaccine acceptance (protective action motivation). These findings are consistent with those suggested in the previous literature [41]. Furthermore, digital media platforms have generated myths, lies, and falsehoods about vaccine speculations [41,42]. This further indicates that misinformation could hinder protective actions and negatively influence one’s motivation to engage in protective behavior [45]. For example, those individuals who perceive the stakeholders or resources (i.e., the polio vaccine) as reliable and trustworthy would be less vulnerable to misinformation.

The findings of this study also prove that religious fatalism inversely moderates the relationship between risk perception and polio vaccine acceptance. Therefore, more fatalistic individuals are less likely to participate in preventive care [46]. Religious beliefs and practices can clash with medical experts’ recommendations and negatively impact a patient’s health, leading to misconceptions in patient awareness and non-compliance with prescribed treatments. In other words, those with fatalistic beliefs tend to be less willing to participate in health-related programs. As a result, such individuals downplay the seriousness of their diseases by refusing treatment.

Moreover, the key barriers and characteristics in the literature are highlighted in Table 5. In sum, this research has clarified that public service advertisements can reduce polio vaccine hesitancy by countering the adverse effect of the barriers in many ways. For example, hypotheses 7a, 7b, and 8 validated past studies’ results describing the obstacles in developing vaccine acceptance. However, the results of H4, H5, and H6 clarified that media contents could effectively vanquish vaccine hesitancy. In this way, the results emphasize that vaccine acceptance can be enhanced by combating these sources and barriers of polio vaccination reluctance in Pakistan. The counter-communication strategies are presented in the next section of this study. The results of H7a and H7b established that public service advertisements could counter the overabundance of misinformation, such as conspiracy beliefs or country of vaccine origin (see Table 5 and Figure 4). Likewise, the results of H8 validated that public service advertisements are better communication tools for diminishing society’s fatalistic views about vaccination (see Table 5 and Figure 3).

### 5.1. Managerial Implications

These findings provide greater and more profound insight for public health practitioners to respond to polio vaccine hesitancy. The study offers practical information to the concerned authorities about the deliberate application of communication resources to minimize barriers such as misinformation, disinformation, and fake news that hinder polio vaccine acceptance. The literature advocates that when barriers create uncertainties about vaccines, the strategic use of media, primarily public service messages, could be used as a remedy to raise awareness among the masses regarding the benefits of vaccines. In the context of the polio vaccine, the development of vaccines has been established as a beneficial product to save people from this disabling and deadly disease. Conversely, polio vaccine hesitancy is considered a barrier that minimizes public acceptance. Therefore, the result of this study provides a valuable strategic communication tool to create awareness about perceived polio vaccine risk, improving the perception of stakeholders and protective perception about polio through the use of public service messages to increase polio vaccine acceptance. The level of religious fatalism could also be decreased by increasing the exposure to media, especially public service messages. Thus, policymakers may use public service messages strategically to counter polio vaccine acceptance barriers, including misinformation, disinformation, fake news, and religious fatalism, in both traditional and new media.

Regarding the societal and digital environmental aspects, this research identified potential barriers, such as religious fatalism and misinformation, hindering the polio vaccine acceptance. People are now living in the digital era, frequently encountering unchecked content on health issues disseminated through digital means. Many of these contents are user generated and contain misleading information and individual standpoints, e.g., religious aspects, on various health issues. These views and misleading information can severely damage mass vaccination campaigns. Thus, this study measured the responses of parents, who are the main stakeholders for a successful vaccination campaign. The results advocated that religious fatalism and misinformation have inverse effects on parents’ acceptance of the polio vaccine. In light of mounting concerns about endemic nations such as Pakistan, these results validated the role of public service advertisements as the critical factor in developing the broader acceptance of polio vaccination campaigns. Likewise, societies with a higher degree of religious fatalism, such as African countries, are also vulnerable to vaccine hesitancy. Policymakers can use this research to plan better communication campaigns to enhance polio vaccine acceptance.

Furthermore, the respondents of this study revealed the tendency of inverse influence from digital misinformation and fake stories mainly containing misleading information about the vaccine country of origin and safety. Moreover, fatalistic religious viewpoints are also revealed as a potential source of mistrust between parents and healthcare authorities. The results found a positive aspect regarding the role of public service advertisement: it can diminish the adverse influences of societal and digital communicative ecology factors. The results provide a policy guideline to develop crisis communication campaigns to counter the misinformation and societal views that can create confusion about the uptake of polio vaccination. These communication campaigns can be more effective in delivering informational support to the stakeholders (parents) by providing them with the technical knowledge and benefits of the polio vaccine to vanquish the polio threat for their children.

### 5.2. Limitations and Future Research

The findings of this study contribute to the body of knowledge by addressing polio vaccine acceptance. Nevertheless, this study has several limitations. First, the study utilized the cross-sectional design that deals with only a snapshot of the phenomenon under investigation, rather than offering a dynamic picture of the phenomenon using longitudinal or experimental designs. Therefore, future studies could utilize longitudinal or experimental designs to track the changing behavior regarding polio vaccine hesitancy or to identify causes of decline in the acceptance of the vaccine. Second, the study investigated the influence of public service messages; future studies could combine the impact of public service messages, news frames, documentaries, etc. Finally, the locus of this study was Pakistan; therefore, the study’s findings can only be generalized to other contexts and countries with considerable caution.

## 6. Conclusions

The development of vaccines has been a significant breakthrough and service to humanity in human history. It has enabled humans to minimize and consequently eradicate the threats of viruses to humanity. However, regardless of the benefits and efficacy of vaccines—including the polio vaccine—public reluctance and skepticism are the most significant challenges to vaccination campaigns across the globe. In this scenario, our findings provide empirical evidence to deal with public reluctance and skepticism through strategic media messages to cultivate the literacy necessary to counter the phenomena that breed polio vaccine reluctance, such as misinformation, disinformation, fake news, and religious fatalism. Put differently, we suggest that the targeted use of public service messages through media dissemination could be an effective strategy to counter polio vaccine acceptance resistance owing to the current flood of misinformation, disinformation, fake news, and religious fatalism, particularly in Pakistan.

## Figures and Tables

**Table 1:** Correlations.

	Mean	PSA	RP	RF	SP	PPA	ID	PAM
PSA	3.25	1						
RP	3.65	0.089	1					
RF	3.56	−0.078	−0.472	1				
SP	3.81	0.187	−0.145	−0.202	1			
PPA	2.78	0.087	0.326	0.506	−0.171	1		
ID	4.09	−0.127	−0.063	0.151	−0.234	−0.072	1	
PAM	3.93	0.054	0.025	0.081	0.252	0.193	0.164	1

**Table 2:** Item standardized weights.

Variables	Estimate
Public Service Advertisements	
PSA1	0.79
PSA2	0.92
PSA3	0.64
Risk Perception	
RP1	0.97
RP2	0.86
RP3	0.76
Stakeholder Perception	
SP1	0.81
SP2	0.89
SP3	0.77
Religious Fatalism	
RF1	0.75
RF2	0.69
RF3	0.68
RF4	0.77
Protection Action Motivation (Polio Vaccine Acceptance)	
PAM1	0.80
PAM2	0.77
PAM3	0.83
Protection Perception Attributes (Dimension: Resource-Related Attributes)	
RA1	0.85
RA2	0.89
RA3	0.87
Protection Perception Attributes (Dimension: Hazard-Related Attributes)	
HA1	0.84
HA2	0.79
Misinformation	
MI1	0.83
MI2	0.85
MI3	0.72

**Table 3:** Validity statistics.

Variables	CR	AVE	PSA	RP	SP	PAM	PPA	RF	MI
PSA	0.830	0.63	0.790						
RP	0.898	0.75	0.158	0.865					
SP	0.864	0.68	0.182	−0.042	0.824				
PAM	0.844	0.64	0.076	0.190	0.33	0.802			
PPA	0.966	0.93	0.146	0.073	0.17	0.202	0.967		
RF	0.815	0.53	0.124	−0.17	−0.24	−0.16	−0.53	0.725	
MI	0.844	0.65	0.105	−0.15	−0.16	−0.23	−0.36	0.505	0.802

**Table 4:** Hypothesis testing.

Direct Influence	β	*p*-Value	T-Vale	Hypothesis
Risk Perception → PAM (Polio Vaccine Acceptance)	0.26	0.001	5.81	H1 Supported
Stakeholder Perception → PAM (Polio Vaccine Acceptance)	0.38	0.001	6.91	H2 Supported
Protective Action Perception → PAM (Polio Vaccine Acceptance)	0.41	0.001	7.18	H3 Supported
PSA → Risk Perception	0.24	0.001	4.25	H4 Supported
PSA → Stakeholder Perception	0.31	0.001	5.98	H5 Supported
PSA → Protective Perception Against Polio	0.37	0.001	6.34	H6 Supported
Misinformation X Protective Perception → PAM	−0.27	0.012	3.57	H7a Supported
Misinformation X Stakeholder Perception → PAM	−0.21	0.031	3.61	H7b Supported
Religious Fatalism X Risk Perception → PAM	−0.18	0.001	4.38	H8 Supported

RPSV = Risk perception about COVID.

**Table 5:** Key barriers in the literature.

Authors	Year	Barriers	Method	Context
Puri [41]	2020	Social Media Disinformation	Survey	Vaccine Hesitancy
Ali [42]	2020	Misinformation	Systematic Review	Positive Psychology
Basch [43]	2017	Vaccination Risks and Reactions	Content Analysis YouTube	Vaccine and Children
Gisondi [44]	2022	Infodemics and Misinformation	Opinion	Vaccine Hesitancy
Ittefaq et al. [9]	2021	Misleading Viral Videos	Commentary	Polio Vaccine
Dettenborn [47]	2004	Religious Fatalism	Cross-Sectional study	Health Behavior
Salazar-Collier [49]	2021	Religious Fatalism	Cross-Sectional study	Health Behavior

## Data Availability

The data supporting this study’s findings are available from the corresponding author upon reasonable request, due to ethical and privacy restrictions.
